# An Alternative Alar Cinch Suture

**Published:** 2010-12-20

**Authors:** Raffaele Rauso, Nicola Freda, Giuseppe Curinga, Claudio Del Pero, Gianpaolo Tartaro

**Affiliations:** ^a^Maxillo-Facial Surgery, Head & Neck Department, II University of Naples, Naples, Italy; ^b^Plastic Surgery, Private Practice, Serravalle, Lucca, Italy; ^c^Plastic Surgery, Civico Hospital, Palermo, Italy; ^d^ORL, Civico Hospital Santa Maria Goretti, Latina, Italy

## Abstract

Nasal widening is commonly associated to maxillary osteotomies, but it is only partially dependent on the amount of skeletal movement. Techniques for controlling lateralization of the ala, including the alar base cinch technique, originally described by Millard, have been well reported by Collins and Epker and later modified by others. In this article, authors report the effect of a new alar cinch suture technique on a sample of 32 patients.

Many studies have shown significant changes in soft tissue nasolabial morphology associated with Le Fort I osteotomy,^1^^-^4 one of which is an increase in the width of the alar base of the nose.

Le Fort I osteotomy alters the proportion of the alar base and widens it, the superior or anterior maxilla movement has the most effect.^5^ If the alar base is widened, the nostril shape is altered. Excessive widening and superior retraction result in an ugly deepening of the alar-cheek groove, making the patient look older. The technique for controlling lateralization of the ala, including the alar base cinch technique, was originally described by Millard^6^ to correct nasal defects in patients with cleft lip, then described by Collins and Epker^7^ for its use in noncleft patients, and later modified by others.^8^,^9^

In this article, authors report the effect of a new alar cinch suture technique on a sample of 32 patients.

## SURGICAL TECHNIQUE

**Figure 1:**
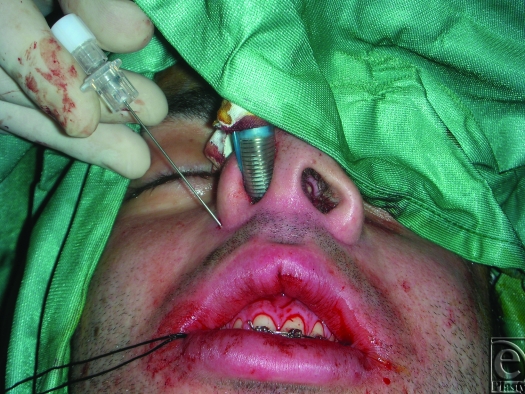
An 18-gauge needle is inserted through the skin at the nasofacial skin fold of the right alar base and exits in the mouth at the fibroareolar tissue.

**Figure 2:**
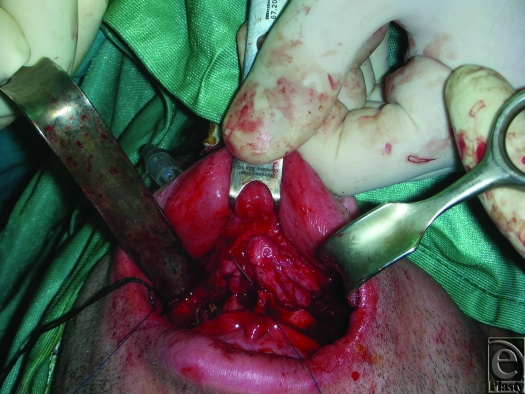
Picture 1. The needle is retracted through the skin point without leaving it, then returned to the oral cavity again in a medial position.

**Figure 3:**
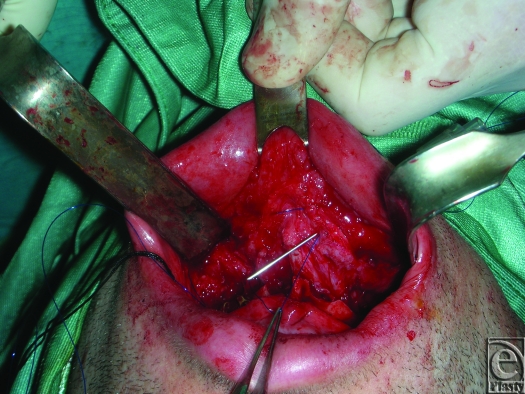
Picture 2. The same procedure is done through the skin point at the other side of the nose.

**Figure 4:**
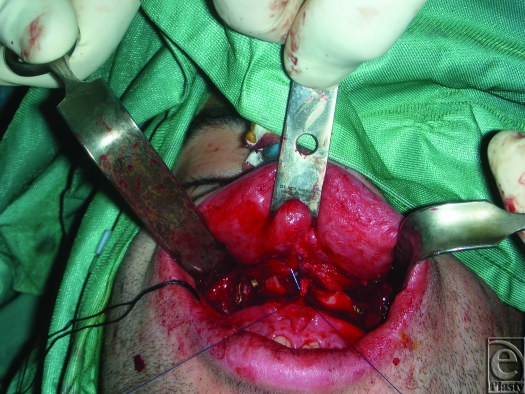
Pictures 3 and 4. The two free ends of the sutures are passed through a hole made in the nasal spine making a knot.

An 18-gauge needle is inserted through the skin and exits at the fibroareolar tissue (Fig 1). A 3/0 nonabsorbable suture without a needle is inserted through the needle from the oral cavity to outside. The needle is retracted through the skin point without leaving it and then returned to the oral cavity again in a medial position (Fig 2; Picture 1). Finally, the needle is retracted from the skin leaving the suture through the soft tissues. The same procedure is done through the skin point at the other side of the nose (Fig 3; Picture 2). The 2 free ends of the sutures are then passed through a hole made in the nasal spine making a knot (Fig 4; Pictures 3 and 4).

## PATIENTS AND METHODS

**Figure 5:**
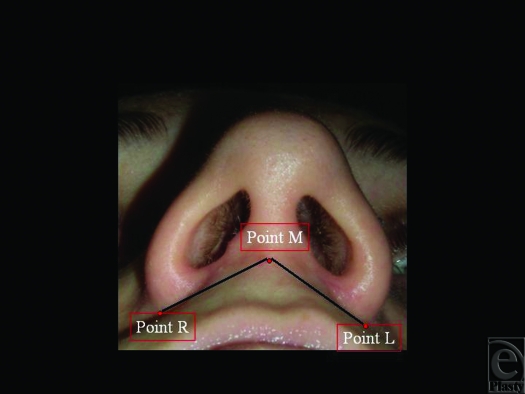
Landmarks of the base of the nose.

Thirty-two patients with skeletal class III facial deformity who had orthognathic operations by the same surgeon were recruited in this study. All the patients had bimaxillary operations, with or without genioplasty. Exclusion criteria were cleft lip, previous nasal operation, and previous or simultaneous additional midfacial operations. In the sample, there were 13 men and 19 women, average age 25.3 years (range, 18.1–37.3 years). The base of the nose was marked with 3 landmarks: the nasofacial skin fold at the left alar base (point L), the middle of the columella (point M), and the nasofacial skin fold at the right alar base (point R) (Fig 5). Distances between points L and M and between points M and R were recorded before, and 1 year after, operation for each patients (Table 1). The nasal base changes were evaluated by the surgeon and the whole equipe. Total follow-up is now of 1 year.

## RESULTS

There were no major or minor complications. Comparing the preoperative and the 6-months postoperative distance between points L and M and points M and R, in 19 of 32 patients it was unchanged; in 13 of 32 patients, it was changed in a rate ranging between ‐2 mm and +3 mm (Table 1).

Aesthetic outcomes were evaluated by the patients, comparing the preoperative photographs with those taken 6 months after surgery; in all the cases, no compliant about nasal base width was recorded.

## DISCUSSION

Nasal widening is commonly associated to maxillary osteotomies, but it is only partially dependent on the amount of skeletal movement. What is more important is the degree of subperiosteal dissection and the amount of soft tissue elevated, that in most surgical techniques involves the total maxilla. The freeing of the facial muscles from the nasolabial area and the anterior nasal spine allows the muscles to retract laterally, which results in flaring, widening, and raising of the base of the nose, which is commonly asymmetrical.

In a recent prospective, randomized, controlled trial study, Howley et al^10^ assessed that the use of the alar cinch suture was effective in controlling the width of the alar base of the nose after Le Fort I osteotomy.

Alar cinching through the vestibular incision used for maxillary osteotomy seems to be a simple and convenient way of narrow the alar base.

In 1993, Loh^8^ proposed a modification of the technique of alar base cinching, to be used in presence of nasotracheal intubation, often used during orthognathic surgery. Indeed, the nasotracheal tube poses 2 problems: the measurement of the alar base may not be accurate because of the distortion of the nostril by the tube; tightening of the cinch sutures is also limited by the tube. Loh's technique differs from the one proposed by Collins and Epker because after performing the classic cinch suture, an 18-gauge needle is used to create a channel from the skin to the fibroareolar tissues, enabling the cinch sutures to be brought outside the face. The 2 free ends of the sutures and the needle hub are temporarily secured to the facial skin with surgical tape. Final tightening of the sutures is done after extubation and with the patient fully awake or even several days after the surgery. Tension is applied to the sutures with the needle hub pressed against the alar base and the skin, thus resulting in narrowing of the alar width. Tension is applied until the desired alar width is achieved, and then a fine artery forcep is used to clamp the suture ends at the point at which they exit the needle hub. This prevents the sutures from sliding back into the tissues. The needle is then pulled out together with the artery forcep that holds the sutures until the blunt end of the needle is seen. Another fine, curved artery forcep is used to clamp the sutures as they enter the blunt end of the needle. The first forcep is then removed together with the needle. The free ends of the sutures are tied into a firm knot against the forcep that hold them together. The sutures are cut short, the forcep is released, and the knot can dig into the tissue channel made by the needle.

This modification is interesting but shows some pitfalls. We think that the procedure performed postoperatively creates a lot of discomfort for the patients; asymmetry due to the knot performed on a side of the nose and not in the midline may result. Finally, a skin infection on the site where the knot submerges under the skin may develop.

In 2002, Shams and Motamedi presented another modification of the alar cinch technique.^9^ Their technique is performed as follows: the fibroareolar tissue of the alar base is located through the circumvestibular incision anteriorly, an appropriate suture is inserted through the incision, engaging the fibroareolar tissues and musculature. The needle is then pulled out of the skin in the inferolateral portion of the alar crease, which has been premarked; it is then reinserted into the mouth through the same puncture site. The suture is pulled back and forth several times until it is embedded under the skin into the dermis to prevent an unsightly dimple. Once the needle enters the mouth through the anterior circumvestibular incision, it is passed through the skin; it is again reinserted into the mouth through the same perforation, and the suture is tied down beneath the nasal aperture in the midline. The only limit is due to the use of a curved needle, which is difficult to insert in the desired direction and pass through the same hole.

Rauso et al^11^ have previously shown that this modification is more effective than the classic technique. The technique presented by the authors shows the advantages of the use of a straight needle passing in the same hole without going out from the skin point, this allows the surgeon to control the narrowing of the alar base, anchoring enough soft tissue avoiding postoperative relapse, and avoiding the risk of skin infection in the area to be narrowed too. We believe that the strength of our modified technique is based on its simplicity and consistency; the midline knot anchored to the hole in the nasal spine allows a more symmetric result and avoids the problem of an infection or a foreign body reaction in the skin.

## Figures and Tables

**Figure:**
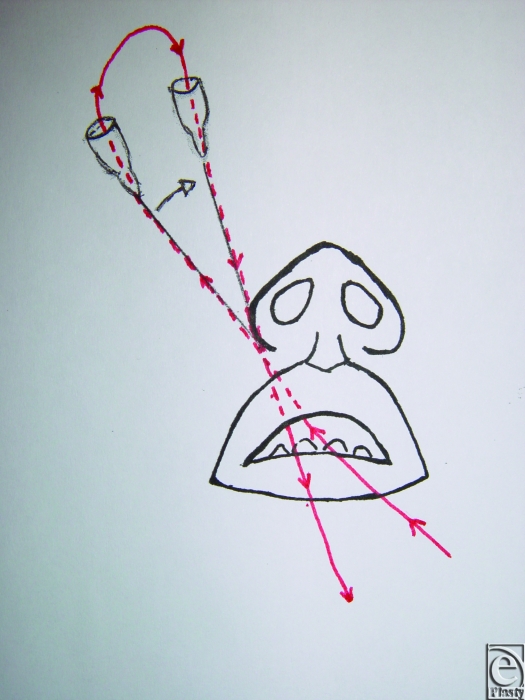
Picture 1.

**Figure:**
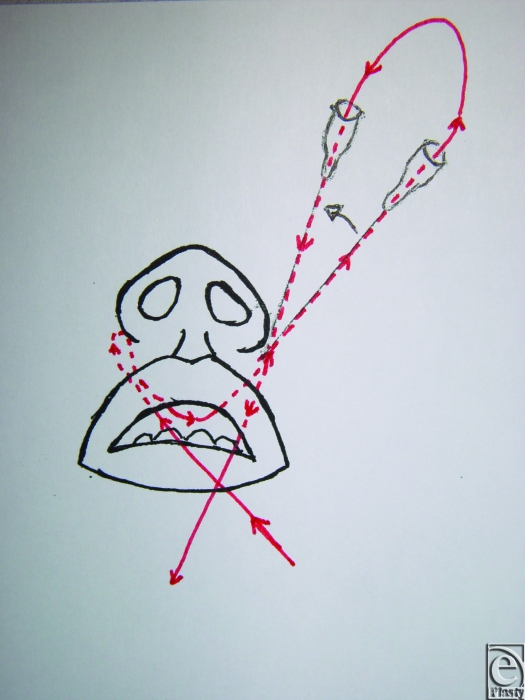
Picture 2.

**Figure:**
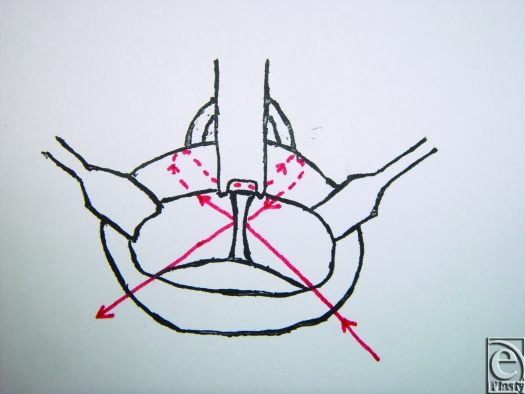
Picture 3.

**Figure:**
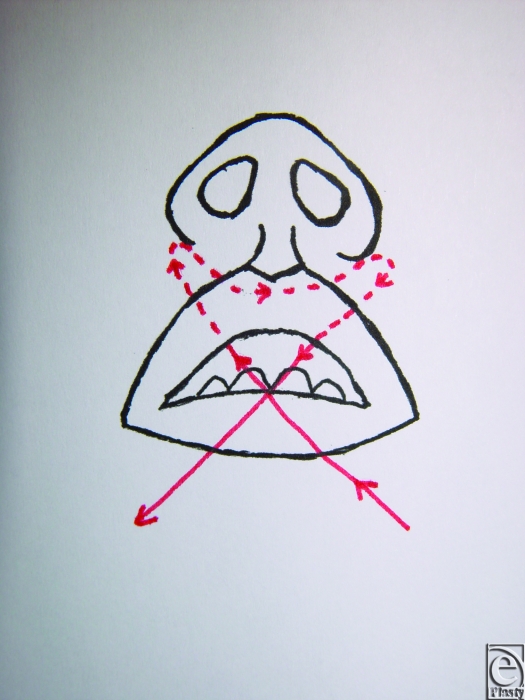
Picture 4.

**Table 1:** Distances (mm) between the left alar base and the columella (L-M) and the columella and the right alar base (M-R) before and 6 months after operation*†

	Before Operation		After Operation			
Patient	L-M, mm	M-R, mm	L-M, mm	M-R, mm	Maxillary	Movements, mm
1	17	18	20	20	6	CCR 2
2	20	20	20	20	3	
3	18	18	18	18	2	
4	19	19	20	20	3	
5	18	16	18	17	4	
6	21	20	21	20	3	
7	18	19	19	20	4	
8	18	18	18	18	2	
9	20	21	20	21	5	
10	17	16	18	18	5	IMP 2
11	21	21	21	21	3	
12	22	20	21	21	1	CR 3
13	16	14	16	14	4	
14	17	17	17	17	3	
15	21	20	21	20	2	
16	18	17	18	17	4	
17	18	18	18	18	3	
18	16	14	18	16	4	IMP 4
19	17	16	17	16	3	
20	19	18	19	18	3	
21	19	19	19	19	5	
22	20	20	21	22	4	CR 2
23	20	20	20	20	6	IMP 2
24	22	21	23	22	2	
25	18	18	18	18	7	CR 2
26	16	19	16	19	2	
27	17	19	18	20	3	
28	19	20	20	21	4	
29	15	16	15	16	3	
30	16	13	16	13	3	
31	18	17	20	19	4	
32	15	16	16	17	5	CCR 2

*CCR indicates counterclockwise rotation; CR, clockwise rotation; IMP, impaction.

†On the right, maxillary movements are listed.
